# Host-Directed Therapeutic Strategies for Tuberculosis

**DOI:** 10.3389/fmed.2017.00171

**Published:** 2017-10-18

**Authors:** Afsal Kolloli, Selvakumar Subbian

**Affiliations:** ^1^Public Health Research Institute (PHRI) at New Jersey Medical School, Rutgers Biomedical and Health Sciences (RBHS), Rutgers University, The State University of New Jersey, Newark, NJ, United States

**Keywords:** tuberculosis, autophagy, host–pathogen interactions, vitamin D, anti-tuberculosis drugs, infant, adjunct therapy-tuberculosis

## Abstract

Tuberculosis (TB), caused by *Mycobacterium tuberculosis* (*Mtb*), remains a leading cause of morbidity and mortality in humans worldwide. Currently, the standard treatment for TB involves multiple antibiotics administered for at least 6 months. Although multiple antibiotics therapy is necessary to prevent the development of drug resistance, the prolonged duration of treatment, combined with toxicity of drugs, contributes to patient non-compliance that can leads to the development of drug-resistant *Mtb* (MDR and XDR) strains. The existence of comorbid conditions, including HIV infection, not only complicates TB treatment but also elevates the mortality rate of patients. These facts underscore the need for the development of new and/or improved TB treatment strategies. Host-directed therapy (HDT) is a new and emerging concept in the treatment of TB, where host response is modulated by treatment with small molecules, with or without adjunct antibiotics, to achieve better control of TB. Unlike antibiotics, HDT drugs act by directly modulating host cell functions; therefore, development of drug resistance by infecting *Mtb* is avoided. Thus, HDT is a promising treatment strategy for the management of MDR- and XDR-TB cases as well as for patients with existing chronic, comorbid conditions such as HIV infection or diabetes. Functionally, HDT drugs fine-tune the antimicrobial activities of host immune cells and limit inflammation and tissue damage associated with TB. However, current knowledge and clinical evidence is insufficient to implement HDT molecules as a stand-alone, without adjunct antibiotics, therapeutic modality to treat any form of TB in humans. In this review, we discuss the recent findings on small molecule HDT agents that target autophagy, vitamin D pathway, and anti-inflammatory response as adjunctive agents along with standard antibiotics for TB therapy. Data from recent publications show that this approach has the potential to improve clinical outcome and can help to reduce treatment duration. Thus, HDT can contribute to global TB control programs by potentially increasing the efficiency of anti-TB treatment.

## Introduction

Tuberculosis (TB) continues to be a major health threat, particularly in developing countries. In 2015, about 10.4 million new cases and 1.4 million deaths were attributed to TB worldwide (1). In addition, nearly a third of the world population is believed to carry latent *Mycobacterium tuberculosis (Mtb)* infection (LTBI) (2). Out of the billions of people with LTBI, about 10% will develop symptomatic, active TB during their lifetime. Thus, these LTBI individuals are a reservoir for potential future active TB cases. The morbidity and mortality due to TB are further accelerated by coinfection with HIV, development of drug-resistant *Mtb* strains, and coexistence of other chronic illness such as diabetes (3–5).

The current TB treatment regimen, implemented by the WHO includes administration of four first-line antibiotics isoniazid (INH), rifampicin (RIF), pyrazinamide (PZA), and ethambutol for 2 months followed by INH and RIF for 4 months. Since *Mtb* can develop resistance more rapidly to individual drugs, a standard TB therapy [directly observed treatment, short-course (DOTs)] with a combination of these four drugs was established in the 1980s. Since then, DOTs have been shown to be effective in achieving microbiological cure in patients with drug-sensitive TB. Although successful compliance of DOTs should not contribute to the emergence of drug resistant *Mtb* strains in these patients, incorrect drug prescription/treatment and patient non-compliance can lead to *Mtb* drug resistance, mostly to INH and RIF, two of the most important/potent first line drugs, resulting in the development of MDR- and XDR-TB cases. Recent epidemiological data have revealed nearly half-million newly diagnosed MDR cases and an additional 100,000 of RIF-mono-resistant TB cases worldwide; about 10% of MDR cases were also found to have XDR (1). The prolonged and complicated anti-TB chemotherapy for MDR- and XDR-cases is not only expensive and not sufficiently effective in achieving the cure but also causes adverse, toxic side effects, challenging patient compliance to treatment. These dire limitations emphasize the need for new treatment and management strategies for both drug-sensitive and drug-resistant TB. In this review article, we discuss the various host-directed therapeutic (HDT) approaches that have gained considerable research interest as an adjunct to antibiotic-based anti-TB treatments.

## Immune Response in TB

Tuberculosis is transmitted through inhalation of *Mtb*-containing aerosol; in about 95% of cases, wherein the tubercle bacilli was inhaled, a primary infection is established. However, progressive, active TB is successfully prevented in >90% of these individuals by the host immune response, resulting in latent *Mtb* infection (LTBI) with no visible symptom of active disease. Individuals with LTBI have 5–10% lifetime risk of developing active TB and host immune suppressing conditions further increases this risk. This underscores the critical role of host innate and adaptive immune response in the control of *Mtb* infection (6). The host immunity to infection is initiated following the uptake of *Mtb* by phagocytes, such as alveolar macrophages and dendritic cells (DCs), in the lower respiratory tract. The interaction between phagocyte pattern recognition receptors and *Mtb* antigens triggers the production of various proinflammatory cytokines, including tumor necrosis factor-α (TNF-α) and interleukin-12 (IL-12) as well as chemokines that recruit and activate other innate and adaptive immune cells from the circulation to the site of infection (7–9). The accumulation of various immune cell types surrounding the infected phagocytes, in response to secreted cytokines and chemokines, results in the formation of granulomas, a hallmark of *Mtb* infection. Although granulomas have been thought to act as a physiological barrier in preventing dissemination of infection and providing a microenvironment that facilitates the interaction between the immune cells and the pathogen, it can also serve as a niche where *Mtb* can thrive and persist (10–12). The fate of intracellular *Mtb* within the phagocytes is determined by various cellular processes, including apoptosis, autophagy, and activation of host defense pathways that produce antimicrobial peptides and reactive oxygen/nitrogen species (13–15). These biological functions of activated macrophages and DCs are capable of killing *Mtb*; however, it is well documented that virulent *Mtb* can prevent the fusion and acidification of phagolysosomal compartments, which are crucial for *Mtb* killing, thus evading the host immune response to survive within these cells (16–18).

While innate immune mechanisms are mostly insufficient to prevent *Mtb* infection from progressing into active disease, phagocytes such as DCs with engulfed *Mtb* and/or antigens can migrate to the regional lymph nodes to prime CD4+ and CD8+ T cells. These activated cells of adaptive immunity establish a Th1 type immune response, marked by induction of interferon-γ (IFN-γ) secretion, which can activate antimicrobial functions and apoptosis of infected macrophages through Fas/Fas ligand interaction (19). In these cells, IFN-γ also enhances the recruitment of CD4+ and cytotoxic T lymphocytes to the site of infection, thus promoting antigen presentation and mycobacterial killing by phagocytes (20, 21).

**Figure 1:**
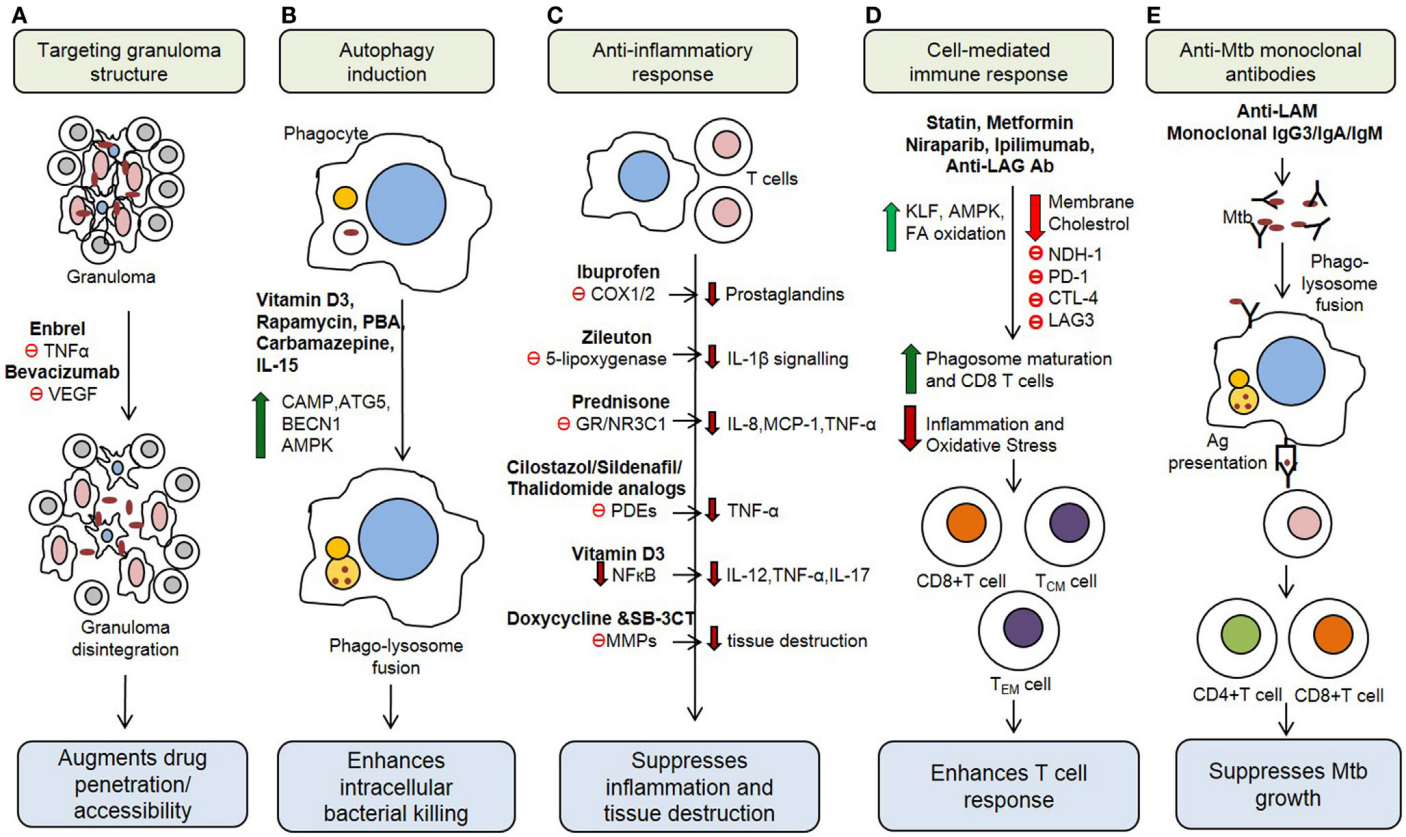
Potential host therapeutic targets against *Mycobacterium tuberculosis*. **(A)** Host-directed therapeutic (HDT) drugs change the integrity of granuloma and enhance drug accessibility. **(B)** Some HDT agents upregulate production of antimicrobial peptides, reactive oxygen and induce autophagy in infected cells. **(C)** HDT drugs suppress proinflammatory responses, which decrease inflammation and tissue damage during active stage of the disease. **(D)** HDT agents regulate cell-mediated immune responses, including antigen-specific T cell responses. **(E)** Monoclonal antibody administration IS other emerging HDT concept for TB treatment. VEGF, vascular endothelial growth factor; PBA, phenylbutyrate; CAMP, cathelicidin antimicrobial peptide; ATG5, autophagy-related protein 5; BECN1, beclin-1; AMPK, AMP-activated protein kinase; COX1/2, cyclooxygenase-1/2; GR, glucocorticoid receptor; PDE, phosphodiesterases; MMPs, matrix metalloproteinases; KLF, Kruppel-like factor; PD-1, programmed cell death 1 receptor; CTLA-4, cytotoxic T-lymphocyte-associated protein 4; LAG3, lymphocyte activation gene 3; LAM, Lipoarabinomannan.

The Th1 immune response to *Mtb* infection is further enhanced by IL-17, a Th17-type cytokine that enhances the secretion of IFN-γ and IL-12 from antigen presenting cells (22). Moreover, natural killer (NK) cells, NK T cells, and CD8+ T cells secrete an array of cytotoxic effector molecules, such as perforin, granulysin, and granzymes that can kill intracellular bacteria by lysing (necrosis) the infected cells (23). However, the elevated production of proinflammatory cytokines, such as TNF-α, IL-1β, IL-6, IL-8, and IFN-α/β, and the cytolytic-T cell response can also cause overt lung inflammation and tissue damage during *Mtb* infection (24–27). This dual effect of host immune activation highlights the need for an HDT treatment strategy that can alleviate host tissue/cell damage by regulating the inflammatory response, while enhancing the anti-*Mtb* defense mechanisms, to improve treatment outcomes. Recent studies have shown that *Mtb* growth can be restricted by modulating several host immune cell functions (28, 29) (Figure 1). This information can be harnessed to develop new treatment strategies against both drug-sensitive and MDR/XDR-TB cases.

On the other hand, HDT strategies that modulate and/or suppress the natural host immune response against *Mtb* infection bears a risk of elevating the proportion of LTBI individuals to reactivate, or accelerate the progression of, TB disease. It has been shown that neutralization of TNF-α led to the reactivation of TB in individuals with LTBI (30) and promote the disease progression to fulminant and disseminated disease (9). Similarly, in the absence of standard anti-TB therapy, administration of anti-TNF-α antibodies exacerbates disease severity in a mouse model of pulmonary TB (31, 32). This possibility cautions the application of HDT as a sole approach for TB treatment and justifies the use of HDT as adjunctive to anti-TB drugs. This review is mainly focused on the adjunctive effect of various HDT agents that modulate various aspects of the immune response, including the production of reactive oxygen species, antimicrobial peptide synthesis, cytokine production, autophagy induction, development of cell-mediated immunity, and boosting immunological memory against *Mtb* infection (Table 1). It should be noted that most, if not all, of the HDTs that have been evaluated as a potential therapy for TB in animal models or humans, as mentioned in the following sections, were administered in combination with existing antimycobacterial antibiotics, as an adjunctive therapy.

**Table 1:** List of host-directed therapeutic agents and their application in tuberculosis treatment.

HDT agent	Mechanism of action	Biological significance	Selected reference
Enbrel	TNF-α neutralization	Disrupts granuloma and reduces lung pathology	Bourigault et al. (33)

Bevacizumab	Neutralizes VGEF	Normalize the vascular structure, decreases the hypoxia, and facilitates the entry of drug in the granuloma	Oehlers et al. (34), Datta et al. (35)

Vitamin D_3_	Induces the production of reactive oxygen and nitrogen intermediates, CAMP and DEFB4	Enhance innate immune responses	Liu et al. (36, 37), Yuk et al. (38), Verway et al. (39)
	Upregulates the expression of *Atg5* and *Beclin-1*	Induces autophagy of infected cells	Campbell and Spector (40)
	Suppresses NF-κB signaling pathways, expression of MMPs proinflammatory cytokines and chemokines	Accelerates the resolution of inflammatory responses during the treatment	Coussens et al. (41), Coussens et al. (42), Song et al. (43)
	Downregulates MHC class II molecules and impairs the CD4 T cell activation, suppresses proliferation of cytotoxic cells, enhance differentiation regulatory T cells	Reduces inflammation and tissue injury caused by exacerbated production of cytotoxic molecules	Imazeki et al. (44), Jeffery et al. (45), Baeke et al. (46), Harishankar et al. (47)

Phenylbutyrate	Histone deacetylases inhibitor, Induces expression of *CAMP, Atg5*, and *Beclin-1* production of reactive oxygen species	Promotes colocalization of LL-37 and LC3-II in autophagosomes and restricts *M. tuberculosis* growth inside the macrophage	van der Does et al. (48), Coussens et al. (49), Rekha et al. (50)

Rapamycin	Inhibits mTOR	Induces autophagy	Corcelle et al. (51)

Gefitinib	Inhibits EGFR	Activates autophagy and decreases in *M. tuberculosis* growth inside the macrophage	Stanley et al. (52)

Carbamazepine	Depletes inositol triphosphate and activates AMPK	Induces autophagy and reduces MDR-TB burden in the lungs and spleen	Schiebler et al. (53)

Aspirin	Enhance the LXA4 production	Activates vitamin D-mediated anti-mycobacterial activities	Tobin et al. (54), Morris et al. (55)

Ibuprofen	Inhibits COX1 and COX2, suppresses prostaglandin H2 production	Regulates TNF-α production and reduces inflammatory pathology	Vilaplana et al. (56)

Zileuton	Inhibits 5-lipoxygenase	Suppresses the production of leukotrienes, augments prostaglandin E2, reduces lung pathology	Mayer-Barber et al. (57)

Prednisone and dexamethasone	Glucocorticoid receptor antagonist	Downregulates production of proinflammatory cytokines	Blum et al. (58), Bilaceroglu et al. (59)

CC-3052, CC-11050, cilostazol, and sildenafil	PDE inhibitors, increase the cAMP levels	Downregulate TNF-α level, inflammation, and lung necrosis	Koo et al. (31), Subbian et al. (60), Maiga et al. (61)

Doxycycline, SB-3CT	Inhibits the expression of MMPs	Reduces the bacterial load in the lung	Walker et al. (62), Majeed et al. (63)

Statin (e.g., simvastatin)	Downregulates production proinflammatory cytokines	Suppresses inflammation and tissue damage	Jain and Ridker (64)

	Decrease in the membrane cholesterol levels	Promotes phagosomal maturation and autophagy, augments tuberculocidal activity of first-line drugs	Parihar et al. (65), Skerry et al. (66)

Niraparib	Inhibits poly(ADP-ribose) polymerase, induces the mitochondrial fatty acid oxidation	Removes oxidative stress, maintain memory CD8 T cell responses and promotes cell-mediated immunity	Pirinen et al. (67)

Resveratrol	Increases the respiratory capacity and regulatory T cell frequency	Reduces oxidative stress and regulates severe inflammation	Beeson et al. (68), Wang et al. (69)

Nivolumab/pembrolizumab	Inhibits the expression of PD-1	Augments CD8+ T cell-mediated immune response	Gros et al. (70), Borch et al. (71)

Infusion of mesenchymal stromal cells	Enhances antigen specific T cells and dendritic cell immune response	Facilitate organ homeostasis and tissue repair	Skrahin et al. (72), Joshi et al. (73)

Adoptive transfer of antigen-specific T cells	Targeted killing of infected cells	Restrict the growth and replication of intra cellular pathogen	Axelsson-Robertson et al. (74)

Supplementation of nebulized IFN-γ	Increases in CD4+ T cell response	Improves response to treatment in cavitary TB patients	Dawson et al. (75)

Antituberculin antibodies-IgG3/mIgA	Reduces pathogenecity of *M. tuberculosis*	Prevent reactivation of TB and decrease bacterial load in the lung	Encinales et al. (76), Balu et al. (77)

## HDT Targeting Granuloma Formation

Tumor necrosis factor-α plays a significant role in the granuloma formation and maintenance of its integrity (78). However, granulomas can restrict the access of antibiotics to the *Mtb* contained in granuloma centers (79, 80), prolonging anti-TB treatment. Neutralization of TNF-α using anti-TNF-α antibody (Enbrel) during *Mtb* infection leads to disruption of granuloma integrity, which augments the bacterial clearance by antibiotic treatment and reduces lung pathology (33) (Table 1; Figure 1). Similarly, a clinical trial has shown the blocking of TNF using etanercept during the initial stage of TB treatment accelerated sputum culture conversion with 25% increase in CD4 cells in HIV-associated TB (81). However, anti-TNF-α antibodies can also suppress host immunity, exacerbating disease severity, as shown in animal models (33, 81).

Granulomas are also characterized by increased expression of vascular endothelial growth factor (VEGF) and angiopoietins (Angs) that promote abnormal angiogenesis and a hypoxic microenvironment (34). Earlier studies have shown an increased level of VEGF-A, VEGF-C, Ang-1, and Ang-2 in pulmonary TB patients as compared to healthy controls (82, 83). Antibody neutralization of VEGF (bevacizumab) or treatment with SU5416 (tyrosine kinase receptor inhibitor), and pazopanib (VEGFR inhibitor) enhanced the efficacy of anti-TB drugs by normalizing the vascular structure and reducing hypoxia; these morphological changes facilitated improved anti-TB drug penetration and killing of *Mtb* in the granuloma (34, 35) (Table 1; Figure 1). Together, these studies suggest that granuloma integrity is a critical factor for bacterial survival and proliferation and that disruption of granulomas may promote drug penetration. Thus, a combination of HDT and anti-TB drugs should help to improve the efficacy and clinical outcome as well as to shorten the duration of treatment.

## HDT Targeting the Vitamin D Pathway to Modulate Immune Response

Immunomodulators such as 1,25-dihydroxyvitamin D_3_ [1,25(OH)_2_D_3_] that upregulate the innate immune functions and regulate inflammatory responses have attracted considerable research interest as HDT for TB (84). 1,25(OH)_2_D_3_ has been shown to induce the production of reactive oxygen and nitrogen intermediates, antimicrobial peptides such as cathelicidin antimicrobial peptide (CAMP) and beta-defensin-4 (DEFB4), whereas it limits iron availability for intracellular bacteria and inhibits the accumulation of lipid droplets in *Mtb*-infected macrophages (36–39, 85–90). Although these studies are promising, clinical trials on vitamin D supplementation therapy revealed contrasting results [(91–93); see below].

The *Mtb*-killing activity of macrophages can be enhanced by a combination of modulating the vitamin D pathway and inhibiting the activity of histone deacetylases (HDACs) (94). HDACs are a group of enzymes that remove acetyl groups from histones; inhibition of HDACs typically promotes gene transcription (95). Phenylbutyrate (PBA) is an HDAC inhibitor, which induces CAMP/LL-37 gene expression in cell lines and suggested to have a role in the treatment of *Mtb* infection (96). Administration of PBA (500 mg) in combination with vitamin D_3_ (5,000 IU) and standard anti-TB drug therapy activated CAMP gene expression and suppressed intracellular *Mtb* growth in MDMs (94, 97) (Table 1; Figure 1). Moreover, an *in vitro* study showed that vitamin D_3_ together with PBA treatment promoted autophagy and differentiation of DCs into a stretched CD14+/CD1a−DC subset and enhanced the production of reactive oxygen species and cathelicidin (48). Interestingly, 1,25(OH)_2_D_3_ upregulates the expression of autophagy-related proteins (ATGs), such as ATG5 and Beclin-1 and augments autophagosome initiation, and its fusion with the lysosome and restricts *Mtb* growth (Table 1; Figure 1) (40, 38, 98). *In vitro* studies demonstrated that treatment of *Mtb*-infected macrophages with vitamin D_3_ plus PBA promotes colocalization of LL-37 and LC3-II proteins in autophagosomes (49, 50). A recent clinical study showed that cotreatment with PBA and vitamin D_3_ as an adjunct to standard chemotherapy reduces *Mtb* growth in MDMs and accelerates clinical recovery compared to the placebo group (97) (Table 2). These studies unveil a potential role of vitamin D_3_ in combination with PBA supplementation in the treatment of TB. However, more clinical studies are necessary to validate the potential of D_3_ and PBA as HDT against drug-resistant TB.

**Table 2:** HDT agents in the clinical trials for TB treatment.

HDT agent	Hypothesis	No. of subjects	Dose	Hypothesis acceptance	Reference
PBA + vitamin D3	PBA + vitD3 enhance recovery in PTB patients.	288	500 mg (PBA) + 5,000 IU (VitD3) daily for 2 months	Yes	Mily et al. (97)

Vitamin D3	i. VitD3 supplementation could augment faster recovery.	259	600,000 IU two doses	Yes	Salahuddin et al. (99)
	ii. VitD3 supplementation improves treatment response in PTB.	199	50,000 IUs thrice weekly for 8 week	No	Tukvadze et al. (100)

Prednisolone/prednisone (PN)	i. Adjunctive prednisolone treatment appraise anti-TB treatment in HIV negative advanced PTB patients.	178	20 mg twice times a day	Yes	Bilaceroglu et al. (59)
	ii. Prednisolone therapy enhances immune response HIV-infected TB patients.	187	2.75 mg/kg for 4 weeks	No	Mayanja-Kizza et al. (101)
	iii. Adjunctive PN treatment enhances sputum culture conversion (meta-analysis).	1806	134 mg/day	Yes	Wallis (102)

Dexamethasone	i. Adjunctive dexamethasone treatment can reduce the risk of disability or death in TBM.	545	Patients were graded and different doses were administered	No	Thwaites et al. (103)

IFN-γ	i. IFN-γ treatment accelerates sputum smear conversion.	5	500 µg three times a week for 1 month	Yes	Condos et al. (104)
	ii. Adjuvant IFN-γ inhalation augments recovery in MDR-TB.	6	Two million IU three times a week for 6 months	No	Koh et al. (105)

rIFN-γ	Adjunct rIFN-γ may reduce pulmonary inflammation and promote earlier sputum clearance.	89	200 µg three times for 16 weeks	Yes	Dawson et al. (75)

IFN-α	Adjunct IFN-α treatment improves treatment response in MDR-TB	7	Three million IU, three times a week for 2 months	No	Giosuè et al. (106)

rIL-2	i. rIL-2 treatment enhances both the immune response and bacterial clearance.	110	225,000 IU twice daily for 30 days	No	Johnson et al. (107)
	ii. Adjunct IL-2 supplementation enhance treatment response in MDR-TB.	50	500,000 IU once every other day at the first, third, fifth, and seventh months	Yes	Shen et al. (108)

Etanercept	TNF blockade suppress inflammatory response and enhance treatment response in HIV-associated TB	16	25 mg, eight doses, twice weekly beginning on day 4 of anti-TB therapy	Yes	Wallis et al. (81)

Mesenchymal stromal cell	Adjunct autologous treatment with bone marrow-derived MSCs might improve clinical outcome in MDR/XDR-TB	30	Single-dose of 1 × 10^6^ MSCs per kg	Yes	Skrahin et al. (72)

*PBA, phenylbutyrate; PTB, pulmonary tuberculosis; HIV, human immunodeficiency virus; IFN, interferon; rIFN-γ, recombinant IFN-γ; MDR, multidrug-resistant; XDR, extensively drug resistant*.

1,25(OH)_2_D_3_ also acts as an anti-inflammatory agent by downregulating the production of proinflammatory cytokines and chemokines, such as IL-6 and IL-12, TNF-α, IL-17, IL-23, MIG, IP-10, MCP-1, by suppressing the NF-κB signaling pathway and augmenting the production of anti-inflammatory cytokines TGF-β1, IL-4, and IL-10 in pulmonary TB (41, 43, 109–112) (Table 1; Figure 1). Moreover, *in vitro* studies have shown that 1,25(OH)_2_D_3_ suppresses both T_H_1 and cytotoxic T cell response, whereas it enhances the differentiation of CD4+ CD25-Foxp3+ regulatory T cells, leading to significant reduction of IFN-γ and IL-17 production in many clinical conditions including TB (44–47, 113–116) (Table 1). Two *in vivo* study conducted in TB patients showed that administration of a high dose of vitamin D (2.5 mg or 600,000 IU) with standard anti-TB drugs improved clinically as well as radiological read-outs, suppressed antigen-stimulated proinflammatory cytokine production, and accelerated the resolution of inflammatory responses during treatment (42, 99) (Table 2). However, a recent study reported that vitamin D supplementation at 1.25 mg (50,000 IU) with standard first-line anti-TB drugs corrected the vitamin D deficiency, without significantly altering the *Mtb* culture conversion rates in pulmonary TB patients (92, 93, 100) (Table 2). Moreover, a cross sectional analysis reported that vitamin D supplementation enhanced sputum conversion only in TB patients with vitamin D receptor “*tt*” genotype (93). This study suggests that polymorphisms in genes that are associated with vitamin D signaling pathways can influence the outcome of host response to TB. These conflicting results may be due to the variation in dose, duration of treatment, stage of disease and/or other factors. In addition, since the imbalance in vitamin D level increases the risk of TB (117), interindividual variation of basal vitamin D level among the TB patients may affect the study result. Hence, further studies of sufficient sample size with proper clinical settings are necessary to understand the beneficial effects of vitamin D supplementation in TB.

## HDT Targeting Autophagy

Autophagy is a lysosomal self-digestion process essential for cellular homeostasis, which can function as an innate defense mechanism during *Mtb* infection (118). In particular, it has been demonstrated that a phagosome containing the bacteria can fuse with an autophagosome and subsequently with the lysosome, resulting in pathogen killing (119). Virulent *Mtb* strains can modulate phagosome membrane by expressing the early-secreted antigen 6 secretion system (120). However, STING (stimulator of the interferon gene), a host protein capable of recognizing extracellular bacterial DNA, promotes delivery of bacteria to autophagosome, facilitating bacterial clearance (120). Autophagy can be induced *via* the inhibition of mammalian target of rapamycin complex 1. Rapamycin is an immunosuppressive drug that inhibits mTOR and induces autophagy (51, 121) (Table 1; Figure 1). However, its application as HDT for TB is limited since rapamycin is metabolized by CYP3A4, a hepatic enzyme that is also induced by RIF, an anti-TB drug. Autophagy can also be induced by inhibiting epidermal growth factor receptor (EGFR)-mediated p38 MAPK signaling pathways. Indeed, gefitinib is an inhibitor of EGFR that activates autophagy, and administration of gefitinib (100 mg/kg) in absence of chemotherapy was shown to decrease intracellular *Mtb* growth in macrophages and in a mouse model (52) (Table 1). Thus, activation of autophagy *via* inhibition of EGFR may be a promising HDT avenue.

Anticonvulsant drugs such as carbamazepine have been shown to stimulate autophagy through inositol triphosphate (IP_3_) depletion and AMP-activated protein kinase activation and kill intracellular *Mtb* in macrophages (53). Moreover, carbamazepine treatment without anti-TB drugs significantly reduced the MDR-TB burden in the lungs and spleen and diminished the inflammatory pulmonary infiltrate in mice (53) (Table 1). These studies suggest that HDT drugs that trigger autophagy induction and promote the intracellular killing of mycobacteria may play a significant role in the management of MDR-TB. In addition, following autophagy, the bacterial degradation products were shown to be loaded on the MHC class II molecule, helping to generate efficient adaptive immunity by stimulating the T cell response against *Mtb* (122).

## HDT Targeting the Inflammatory Response

The balance between pro- and anti-inflammatory responses is a critical factor in determining the fate of initial *Mtb* infection. Indeed, a shift toward either aggressive proinflammatory or aggressive anti-inflammatory cytokine response may lead to poor bacterial control and development of active disease (11, 123). Host inflammatory balance is influenced by the level of lipoxin A4 (LPX4) and leukotriene B4 (LTB4): increased production of LPX4 helps to maintain inflammatory balance and play a pivotal role in the control of TB progression (54), while LTB4 causes hyperinflammation and increases disease severity (24). The treatment with aspirin (acetylsalicylic acid), an anti-inflammatory drug, enhances LXA4 production, which in turn suppresses neutrophil migration and TNF-α production, thereby regulating inflammatory pathology during mycobacterial infection, as has been suggested for tuberculous meningitis (TBM) (24, 55) (Table 1).

Prostaglandin and thromboxane are vasoconstrictors that facilitate platelet aggregation and regulate inflammation. Ibuprofen, an immunomodulatory drug that inhibits cyclooxygenase-1 (COX1) and COX2 and suppresses prostaglandin H2 and thromboxane production (124). Ibuprofen treatment without standard anti-TB drugs has been shown to reduce *Mtb* load and diminish inflammatory lung pathology in a murine model of active TB (56) (Table 1; Figure 1). Furthermore, an *in vitro* study reported that indomethacin, a non-steroidal anti-inflammatory drug that inhibits COX1/2 and regulates uncontrolled proliferation of CD4+, CD8+, and regulatory T cells contributing to TB pathogenesis (125) (Table 1). This suggests a potential role for indomethacin in alleviating the inflammatory response during active TB; however, the efficacy of these drugs, as adjunct to antibiotics, has yet to be validated under *in vivo* conditions.

The inflammatory response can also be regulated by down-modulating the activity of lipoxygenase (126). Zileuton is an inhibitor of the 5-lipoxygenase pathway, which suppresses the production of leukotrienes and downregulates inflammation, thus improving airway function in asthmatics (127). Zileuton treatment with or without anti-TB drugs modulates IL-1β-mediated signaling pathways, controls exacerbated inflammation by regulating type 1 IFN production, augments prostaglandin E2 level, and significantly reduces *Mtb* burden and lung pathology in a mice model of pulmonary TB (57) (Table 1; Figure 1). However, this compound has not been validated in other, more relevant-to-human models of TB.

Targeting immune response pathways downstream of the glucocorticoid receptor (GR) is another HDT strategy to control aggressive inflammation. Administration of corticosteroids, such as prednisone and dexamethasone (GR agonists), has been shown to ameliorate the proinflammatory response during *Mtb* infection by significantly downregulating the expression of IL-6, IL-8, MCP-1, and TNF-α (58, 128) (Table 1; Figure 1). Corticosteroids that bind to GR have been tested as an adjunct to standard antibiotic therapy in various forms of TB such as pleural effusion, TBM and pericarditis and TB-IRIS in HIV coinfected patients (129). Although earlier studies with adjunct corticosteroid treatment found some beneficial effects such as improvement in lung radiography and pulmonary function at the initial stage of disease, these studies did not find any significant difference between anti-TB drugs with and without the HDT drug in long term studies (130). A recent meta-regression analysis by Wallis reported that corticosteroid with standard TB chemotherapy reduces the proportion of positive sputum culture from 15 to 2% (102) (Table 2). It has been shown that corticosteroid plus anti-TB treatment reduces the mortality rate among TBM cases (103, 131) but did not prevent the severe disability associated with this disease (103) (Table 2). The corticosteroid supplementation as adjunct to antibiotics have also been reported to be beneficial in patients with tuberculous pericarditis (132); however, more studies are required to reach a solid conclusion. In addition, supplementation with corticosteroid alone was shown to have no significant improvement, compared to treatment with antibiotics alone, in PTB patients (133). Similarly, prednisolone therapy (2.75 mg/kg for 4 weeks) in 187 HIV-TB coinfected patients did not show significant beneficial effect other than mild improvement in CD4+ T cell counts (101) (Table 2). In contrast, when prednisone was used as adjunct therapy in combination with standard anti-TB drugs, enhanced radiographic regression, and decreased bacillary load in advanced PTB patients (59, 134) (Table 2). However, detailed studies are required to corroborate the role of prednisone as an adjunct in the treatment of TB.

Inflammation can also be controlled by regulating the activity of phosphodiesterases (PDEs), a group of enzymes that hydrolyze cyclic adenosine and guanosine monophosphates to AMP (135). Using PDE4 inhibitors, such as CC-3052 (25 mg/kg body weight) and CC-11050 (25 or 50 mg/kg body weight), as adjunct to isoniazid, we have shown significantly reduced macrophage activation, expression of TNF-α, lung fibrosis and necrosis, and tissue bacterial load in a mouse and rabbit models of pulmonary TB (31, 60, 136, 137). Similarly, administration of a PDE3 inhibitor (cilostazol) (10 mg/kg body weight) and a PDE5 inhibitor (sildenafil) (10 mg/kg body weight) together with standard TB treatment accelerates bacterial clearance from the lungs and reduces treatment duration by 1 month in a mouse model (61, 138) (Table 1; Figure 1). Thus, adjunct PDE inhibitors with anti-TB chemotherapy may help shorten treatment duration and improve treatment outcome.

During the active stage of infection, *Mtb* induces the expression of various matrix metalloproteinases (MMPs), such as MMP-1, MMP-3, MMP-7, MMP-9, and MMP-10, from lung epithelial cells and macrophages (139). MMPs degrade the pulmonary extracellular matrix, exacerbate the inflammation, and contribute to tissue damage and pulmonary cavitation in TB patients (140, 141). Hence, the inhibition of MMP activity is a promising HDT strategy to alleviate inflammatory pathology in patients. Doxycycline, a tetracycline antibiotic, was shown to suppress the expression of MMP-1, MMP-3, MMP-9, and TNF-α in *Mtb*-infected macrophages and epithelial cells by inhibiting MMP promoter activation (136) (Table 1; Figure 1). Moreover, doxycycline treatment significantly reduced the lung bacterial burden in guinea pigs in a dose-dependent manner (62). However, this study did not investigate the effect of adjunctive doxycycline treatment with standard anti-TB chemotherapy. Another study reported that administration of SB-3CT; an inhibitor of MMPs, along with conventional anti-TBM drugs significantly suppressed the expression of MMP-9 and enhanced *Mtb* clearance in a mouse model of TBM (63).

## HDT Targeting Cell-Mediated Immunity

Cell-mediated immunity is the central part of the adaptive immune response during *Mtb* infection (142), and modulation of the cell-mediated immune response is a promising HDT approach for TB treatment. Several molecules have been identified for their ability to modulate the cell-mediated immune response. Statin is a well-known inhibitor of 3-hydroxy-3-methylglutaryl-CoA that reduces serum LDL cholesterol level in humans (143). In addition, statin acts as a potent anti-inflammatory agent, regulating inflammation and reducing tissue damage in patients with sepsis and pneumonia (144, 145). It activates transcription factor Kruppel-like factor (KLF) and downregulates expression of TNF-α, IL-1β, IL-6, and IL-8 from lymphocytes (64). Statin also decreases IFN-γ production, downregulates the expression of MHC class II molecules, and impairs the CD4 T cell activation (146). A murine model TB study revealed that statin treatment significantly reduces lung bacterial load and disease pathology and this effect was mainly due to a decrease in the membrane cholesterol levels, which promotes phagosomal maturation/acidification and autophagy (65) (Table 1; Figure 1). Moreover, administration of simvastatin augments the anti-TB activities of first-line drugs INH, RIF and PZA in a mouse model (66). Moreover, epidemiological studies have reported a reduced frequency of active TB incidence in statin users compared to non-statin users (147, 148), suggesting that statin therapy lowers the risk of active TB development.

The poly(ADP-ribose) polymerase (PARP) inhibitor; niraparib enhances mitochondrial fatty acid oxidation and helps to alleviate oxidative stress in immune cells (67). Since mitochondrial fatty acid oxidation helps to maintain memory CD8 T cell responses (149), PARP inhibitors may promote the cell-mediated immune response during *Mtb* infection (Table 1; Figure 1). However, more studies are required to validate the role this drug in TB treatment. The nutraceutical resveratrol (*trans*-3,5,4′-trihydroxystilbene) promotes mitochondrial biogenesis and reduces oxidative stress by increasing the cellular respiratory capacity (68). In addition, resveratrol also regulates severe inflammation by increasing regulatory T cell frequency and apoptosis of activated CD8 T cells (69, 150), which may help to control the aggressive inflammatory response and tissue damage during the active stage of TB (Table 1). Inhibitory receptors, such as programmed cell death 1 (PD-1), promote apoptosis of activated T cells, whereas its regulation is critical for differentiation as well as the proliferation of regulatory T cells. Immune checkpoint inhibitors, including nivolumab/pembrolizumab, inhibit the expression of PD-1 on CD8+ T cells and augment cell-mediated immune responses (70, 71). Similarly, cytotoxic T-lymphocyte-associated protein 4 (CTLA-4), expressed by activated T cells, binds to CD80 and CD86 on antigen-presenting cells and transmits inhibitory signal (151). Ipilimumab, a CTLA-4 inhibitor, regulates the immune suppression mediated by CTLA-4 and promotes CD8+ T cell-mediated immune function (152) (Table 1; Figure 1). Further, *in vivo* studies are needed to understand the therapeutic effect of these drugs. The cell-mediated immune response is also enhanced by blocking the activity of lymphocyte activation gene 3 (LAG3), a receptor expressed mainly by regulatory T cells. LAG3 interacts with class II MHC molecules and downregulates CD4+ antigen-specific immune response, their proliferation, activation and homeostasis (153). Another study reported that LAG3 expression more specific to *Mtb* infection and this is coincides with bacterial burden and disease progression (154). This suggests that inhibition of LAG3 function using anti-LAG3 antibody upregulates T cell-mediated immune response and reduces bacterial burden in the lungs (Table 1; Figure 1). In sum, there are several approaches that can be exploited as an adjunct therapy to augment cell-mediated adaptive immune response during *Mtb* infection.

## Immune Cell Therapy as HDT for TB

Direct transfer of immune cells, such as antigen-specific T cells, is an emerging HDT strategy for the management of cancer and infectious diseases (155, 156). Similarly, direct administration of mesenchymal stromal cells (MSCs) and antigen-specific T cells has been evaluated for TB treatment (157) (Table 1). MSCs are tissue resident, multipotent stromal cells that facilitate organ homeostasis and tissue repair following infection and/or inflammation. Treatment of MDR and XDR TB patients with a single dose autologous bone marrow derived MSCs as an adjunct with standard drug regimens enhances T cells and DC immune response specific to *Mtb* antigen (72, 73) (Table 2). In addition, fine-tuning of the T cell-mediated immune response to *Mtb* is a new and emerging concept in HDT (158). For instance, it has been shown that the CD8+ T cell response can be shaped by specific *Mtb* epitopes (74) (Table 1). Thus, the adoptive transfer of activated T cells, such as CD8+ cells, has the potential to be a new promising strategy in the treatment of MDR and/or XDR-TB cases.

## Cytokine Supplementation as HDT for TB

Interferon-γ is an important cytokine that activates innate immune functions and mediates antigen-specific T cell immunity in response to *Mtb* infection (142, 159). Earlier studies reported that inhalation of aerosolized IFN-γ in MDR-TB patients (500 µg, three times a week for 1 month) is associated with sputum smear conversion and radiological improvements (104, 160). However, another study reported that IFN-γ treatment at two million IU, administered three times a week for 6 months, in six MDR-TB patients had no effect on culture conversion rate in long term treatment (105) (Table 2). A randomized controlled clinical study has shown that aerosolized recombinant IFN-γ as an adjunct to standard anti-TB therapy suppressed the production of proinflammatory cytokines, such as IL-1β, IL-6, and IL-8 (75). Furthermore, recombinant IFN-γ supplementation therapy either by nebulization or by subcutaneous injection (200 µg three times per week over 4 months) elevated the CD4+ lymphocytes’ response to purified protein derivative and enhanced *Mtb* clearance in sputum, thus improving the response to treatment in cavitary-TB patients (75) (Tables 1 and 2). While these results are promising, further studies are required to validate the beneficial effect of IFN-γ supplementation as adjuvant therapy in patients with poor responsiveness to anti-TB therapy and in MDR-TB patients. Similarly, a study investigating whether adjunct IFN-α supplementation can improve the anti-TB treatment response among MDR-TB patients did not find significant clinical improvement (106) (Table 2). IL-2 is another important cytokine that is required for the proliferation of antigen-specific CD4+ and CD8+ T cells during *Mtb* infection (161). A randomized controlled clinical trial to test the efficacy of adjunctive IL-2 supplementation with standard anti-TB therapy did not show significant clinical benefits, compared to treatment with antibiotics without IL-2 (107). Interestingly, in a recent clinical study carried out with 50 MDR-TB patients, adjunct supplementation of recombinant human IL-2 (500,000 IU in alternative days and months up to 7 months) in improved immunity status and sputum smear conversion (108) (Table 2). These observations not only highlight the complex roles of cytokines in regulating immune cell functions but also the potential of modulating cytokine activities as HDT for TB treatment.

## Anti-*Mtb* Antibodies as HDT for TB

The role of B cell-mediated humoral immunity in shaping the host response to *Mtb* has been an active area of research for the past several years (162). Recent studies have shown that B cells regulate neutrophil infiltration by modulating IL-17 response and control inflammation during *Mtb* infection in mouse lungs (163). Furthermore, FcR-mediated phagocytosis of *Mtb* was shown to enhance phagolysosomal fusion and mycopeptide presentation to T cells, thus augmenting the Th1 response (164, 165). In addition, other studies reported that precoating of bacilli with serum rich in anti-LAM antibodies enhances phagolysosomal fusion and increases the abundance of IFN-γ-expressing CD4+ and CD8+ T cells (166, 167) (Table 1; Figure 1). It has also been reported that high levels of anti-*Mtb* IgG3 antibodies prevent reactivation of TB in high-risk individuals (76). Another study showed a significantly decreased bacterial load in the lungs of *Mtb*-infected mice following intranasal administration of human gamma globulin (168). Similarly, passive administration of a combination of human monoclonal IgAs against *Mtb* antigens in combination with IFN-γ has been shown to protect against subsequent *Mtb* infection in a mouse model (77). Together, these results highlight the potential of *Mtb* antigen-specific antibodies as adjunctive vaccine candidates and indicate that passive transfer of these antibodies with/without anti-TB therapy can be a novel HDT against *Mtb* infection (169). However, further detailed studies are required to validate these approaches.

## Summary and Conclusion

Investigators are actively searching for new treatment strategies for better management of TB and to prevent the emergence of MDR and XDR TB. Targeting and manipulating host factors impacted by *Mtb* can be used for HDT to control infection and dissemination within/outside the lung. HDT is an emerging strategy, where the bacterial infection is controlled primarily by restoring the impaired host immune responses. Small molecules and biological agents that enhance macrophage antimicrobial activities, induce autophagy, and alleviate excessive inflammation have been shown to be promising candidates for HDT targeting both drug-sensitive and -resistant TB. Since HDT drugs target host cell functions, infecting *Mtb* strains are not expected to develop resistance against these drugs. In addition, HDT agents that have the potential to reduce lung matrix destruction can be beneficial in restoring lung function following the treatment of cavitary TB and Immune Reconstitution Inflammatory Syndrome associated with HIV infection. Multiple studies suggest that adjunctive HDT with standard chemotherapy has the potential to accelerate bacillary clearance and disease pathology, thus enhancing patient response to treatment, improving lung function, reducing drug toxicity, helping prevent the emergence of drug-resistant TB, and shortening treatment duration. While most of the HDT agents tested have shown promising results in *in vitro* studies and in animal models, clinical trials to evaluate the implication of these HDT agents have either failed to reproduce similar beneficial effects as model systems or revealed controversial findings. There are several factors that may contribute to the contrasting results among these studies, such as the number and nature of study subjects (under powered studies leads to misinterpretation) and their age, variation in dose selection, different life style of study subjects including food habitat, smoking, and alcohol consumption etc. In addition, progression of *Mtb* infection into active disease is influenced by several other factors such as coinfection, immune suppression, metabolic disorders, ethnicity, malnutrition, the genetic makeup of individuals that reflects in genetic polymorphisms associated with susceptibility/resistance to infection (3, 4). Therefore, future clinical studies with the implementation of carefully designed clinical trials with more meticulous inclusion and exclusion criteria are warranted to validate the efficiency of existing HDTs and to identify new HDT agents that can target multiple biological host functions. These HDTs, as adjunct to existing/new antibiotics, can potentially improve the quality of treatment for the millions of patients suffering from various forms of TB worldwide.

## Author Contributions

SS conceived the concept. AK and SS wrote/edited the manuscript and agreed for submission.

## Conflict of Interest Statement

The authors declare that the research was conducted in the absence of any commercial or financial relationships that could be construed as a potential conflict of interest.
